# Efficacy and safety of paranasal sinus balloon catheter dilation in pediatric chronic rhinosinusitis: a systematic review

**DOI:** 10.1186/s40463-020-00463-0

**Published:** 2020-09-29

**Authors:** Ahmad A. Mirza, Hatim Y. Shawli, Talal A. Alandejani, Sattam M. Aljuaid, Mahmoud Alreefi, Razan A. Basonbul, Shahad K. Alhomaiani, Banan A. Althobaity, Dhuha A. Alhumaidi, Faisal Zawawi

**Affiliations:** 1 Department of Otolaryngology, Head and Neck Surgery, Faculty of Medicine in Rabigh, King Abdulaziz University, P.O Box 80205, Jeddah, 21589 Saudi Arabia; 2 Department of Otolaryngology, Head and Neck Surgery, Faculty of Medicine, King Abdulaziz University, Jeddah, Saudi Arabia; 3 Department of Otolaryngology, Head and Neck Surgery, Faculty of Medicine, University of Jeddah, Jeddah, Saudi Arabia; 4 Department of Surgery-Division of Otolaryngology, King Saud bin Abdulaziz University for Health Sciences, Jeddah, Saudi Arabia; 5 Department of Surgery-Division of Otolaryngology, Ministry of the National Guard – Health Affairs, Jeddah, Saudi Arabia; 6 King Abdullah International Medical Research Center, Jeddah, Saudi Arabia; 7 Department of Otolaryngology, Head and Neck Surgery, King Abdulaziz Specialist Hospital, Taif, Saudi Arabia; 8 College of Medicine, Taif University, Taif, Saudi Arabia

**Keywords:** Sinusitis, Pediatrics, Child, Balloon catheter dilation, Systematic review

## Abstract

**Objective:**

Chronic rhinosinusitis (CRS) negatively affects quality of life (QoL), and balloon catheter sinuplasty (BCS) has shown good outcomes in adult patients. However, there has not been much research on the effects of BCS on pediatric patients. The objective of this review is to systematically assess the literature for studies demonstrating the effectiveness and safety of BCS in pediatric CRS patients.

**Data sources:**

PubMed, Embase and Cochrane Library.

**Study selection:**

We followed the Preferred Reporting Items for Systematic Reviews and Meta-Analysis recommendations (PRISMA) to conduct our study. Observational- and interventional-based studies reporting efficacy and/or side effects of BCS among pediatric populations were included. Efficacy was evaluated by clinically reliable measures including Sino-Nasal 5 (SN-5) QoL scale. Antibiotic usage and revision surgery were also evaluated.

**Data extraction:**

Articles were screened, and data were obtained. Study design, sample size and demographics, treated sinuses, criteria of inclusion, adjunct procedure(s), follow-up time, and outcomes measured were reported.

**Main findings:**

Out of 112 articles identified, 10 articles were included: two interventional controlled trials and eight observational studies. All studies evaluating QoL by SN-5 showed a remarkable reduction in SN-5 score postoperatively. Improvement in the computed tomography (CT) and endoscopic findings for up to 1 year after operation was reported. Furthermore, the majority of patinets treated with BCS did not recieve any course of sinusitis-indicated antibiotics during long-term follow-up, and they had low surgical revision rates. Minor side effects were reported, most commonly synechia.

**Conclusion:**

Available evidence suggests that BCS is safe and effective for the treatment of CRS in pediatric patients. Future randomized controlled studies with large sample size are warranted. Such studies can further determine the efficacy of BCS in managing children with CRS.

## Background

Chronic rhinosinusitis (CRS) negatively affects quality of life (QoL) and significantly impacts the social function of patients [1]. Nearly 2 million of school-aged children in the USA are estimated to have CRS, leading to expenses of more than 1.8 billion dollars per year [2]. A yearly disease incidence of 2.1% was found among children who visited ambulatory healthcare clinics [3]. In contrast to acute rhinosinusitis, CRS persists for longer than 12 weeks in duration [4]. To diagnose a patient with CRS, a set of criteria must be considered. These criteria can be summarized into subjective symptoms and objective endoscopic or imaging findings [5].

Given the effect of CRS on QoL and the financial burden it places on patients and the healthcare system, there is great interest in optimizing available treatment options and further investigating new modalities of treatment. Treatment of CRS is classified into medical and surgical regimens. The first line of CRS treatment is medical therapy, which includes nasal saline irrigation or sprays, a systemic course of oral antibiotics, and nasal steroid sprays. If the symptoms persist, surgery is indicated [6].

A number of medical treatments have been introduced and become evidence-based modalities for pediatric CRS. The use of nasal saline irrigation in pediatric patients was supported by the European position paper on rhinosinusitis and nasal polyps (EPOS) guideline [7]. Saline irrigation has a safety profile in children, and compliant children were found to have greater symptomatic relief and lesser need for surgery [8]. Nasal steroid is another medical regimen for pediatric CRS. It has been recommended as a part of the first line treatment in pediatric CRS, owing to its utility in adult CRS and pediatric allergic rhinitis, and its favorable safety outcome [7, 9–12]. Although systemic steroid was found to improve clinical symptoms and radiological scores, its use is limited in pediatric populations because of the lack of evidence, and safety was a big concern in the literature [13]. Ancillary medical treatments such as antihistamine, decongestants and anti-reflux therapies are not routinely recommended, unless the patient has a concomitant condition such as allergic rhinitis or gastroesophageal reflux disease [7].

Surgical options in pediatric CRS are indicated when the maximal medical therapies fail [10]. There is a broad consensus that adenoidectomy is the first surgical option for children up to age 12 years, with success rates of 40 to 69% [4, 5, 14–18]. Due to a lack of supporting evidence, it was unable to recommend adenoidectomy beyond age 12. The addition of maxillary sinus irrigation to adeniodectomy provided improvement in 87.5% of the treatment group compared to 60.7% of adenoidectomy-alone group [19]. Therefore, EPOS recommends that adenoidectomy with or without antral irrigation as the first intervention to perform in children with CRS [7]. Given the relatively undeveloped sinuses in young children and the role of adenoid hypertrophy in pediatric CRS, endoscopic sinus surgery (ESS) in young children has a limited role [5]. For older children, ESS is safe and a potentially effective approach in CRS that is refractory to medical management and adenoidectomy [7].

Balloon catheter sinuplasty (BCS), an FDA approved device, has been an available option to treat CRS in adult patients since 2006. BCS is a procedure that involves using a guidewire within the sinus guide catheter to reach the target sinus ostium, then gently advancing the balloon catheter through the inflamed ostium. Once the balloon is placed within the ostium, the balloon is inflated under high pressure (up to 12 atm) leading to microfractures and ostium dilatation of 4-7 mm [20, 21]. Consequently, BCS restores normal sinus ventilation without causing significant damage to the surrounding tissues [22]. In contrast to ESS, where serious complications such as hemorrhage, meningitis, adhesions, and orbital complications have been reported, BCS has demonstrated its safety in multiple studies including prospective long-term studies [23–25]. Furthermore, BCS can be performed in an office setting, as well as in an operating theatre [26].

Current evidence supports the use of BCS in adult CRS patients [27]. There is, however, no current agreement whether BCS is safe or efficacious in treating children with CRS. Since EPOS 2012, a number of studies have been carried out to determine the efficacy of BCS in pediatric CRS [24, 25, 28–33]. The majority of these studies were pooled in a previous meta-analysis [34], demonstrating that BCS can significantly improve CRS symptoms among the pediatric group using a parent-reported scale, Sino-Nasal 5 (SN-5) QoL questionnaire [24, 28, 29, 35, 36]. However, this previous review lacks randomized controlled trials and studies with long-term follow-ups. Additionally, the safety of BCS was not discussed in the previous review.

Therefore, we conducted this systematic review of the efficacy and safety of balloon sinuplasty in pediatric CRS patients. We gained insightful data on long-term outcomes, and we used reliable objective parameters to evalaute the efficacy of BCS, i.e. antibiotic usage and surgery revision rates.

## Methods

Our review was conducted in accordance to the Preferred Reporting Items for Systematic Reviews and Meta-Analysis Protocol (PRISMA-P) statement [37].

### Information sources and search strategy

We carried out a systematic search on November 17, 2019 through three major databases: PubMed, Embase and Cochrane library (CENTRAL). No time or language restrictions were placed in our search. In our search, we used controlled vocabularies (MeSH terms and Emtree). In PubMed we followed the search: (balloon [All Fields] OR sinuplasty [All Fields]) AND (“sinusitis”[MeSH Terms] OR “sinusitis”[All Fields] OR rhinosinusitis [All Fields]) AND (“pediatrics”[MeSH Terms] OR “pediatrics”[All Fields] OR “pediatric”[All Fields] OR “child”[MeSH Terms] OR “child”[All Fields] OR “children”[All Fields]). In Embase, a comprehensive search strategy was applied: ((‘sinusitis’/exp. OR sinusitis) OR (‘rhinosinusitis’/exp. OR rhinosinusitis) OR (‘paranasal sinus disease’/exp. OR ‘paranasal sinus disease’)) AND (sinuplasty OR (‘balloon dilatation’/exp. OR ‘balloon dilatation’) OR (‘balloon catheter’/exp. OR ‘balloon catheter’)) AND ((‘pediatrics’/exp. OR pediatrics) OR (‘child’/exp. OR child). Due to a limited number of related studies in Cochrane library, we chose to use a single search word (“sinuplasty”). Reference lists of the included studies were also screened for relevance.

### Eligibility criteria

The PICOS framework was adopted in this review where we included pediatric or adolescent patients (patients 18 years old and under) who were eligible for balloon sinuplasty with a clinical diagnosis of CRS refractory to medical treatments. The comparator was any alternative medical or surgical methods, or none. The outcomes were classified as:

1. Primary outcome: QoL by using SN-5 or Visual analogue scale (VAS)
2. Secondary outcomes:
  § Revision rate, which referred to the number of children requiring adenoidectomy, BCS, or ESS after primary BCS.
  § Antibiotic usage, that was defined as the number of sinusitis-indicated courses of antibiotics after BCS.
  § Side effects of the procedure.

Randomised controlled trials and observational studies that reported efficacy and/or side effects were included. Conference abstracts, letter to editors, commentaries, and review articles were excluded.

### Study selection

Two review authors (A.M. and S.A.) independently screened the titles and abstracts of identified studies and excluded clearly irrelevant articles. In the second phase, the same two reviewers assessed the full texts of remaining studies using the aforementioned eligibility criteria. In case of any ambiguity, we consulted a senior review author. Author (F.Z.) finally reviewed all selected articles and approved their inclusion.

### Data collection process

Data were extracted by the first author (A.M.) and re-checked by another (S.A.). The following data were extracted from each study: author name, study design, sample size, demographics, treated sinuses, inclusion criteria, adjunct procedure(s), follow-up time and results of QoL assessment, revision rate, antibiotic use, adverse events and other reported outcomes. For the QoL measure, mean and standard deviation (SD) of SN-5 and VAS score of treatment arm (BCS) were extracted, from each study, for both pre- and postoperative periods. Improvement of SN-5 score as described by Kay and Rosenfeld [1] was also extracted and reported. This clinical improvement was defined as a 0.5 decrease or more in SN-5 score.

### Risk of bias assessment

Risk of bias of the included studies was assessed using the Cochrane Collaboration’s Tools for risk of bias assessment. For randomized trials, we elected to use the revised Risk of Bias assessment tool (RoB2) [38]. This tool evaluated various types of bias that may occur in the process of randomization and post-intervention domains. The response to each domain was either “low risk of bias”, “some concerns” or “high risk of bias”. The overall risk of bias for each study was judged at “low risk of bias” when all domains of bias were labeled “low”. The study was judged at “some concerns” when there were “some concerns” at least in one domain. The study was judged at “high risk of bias” when at least one domain was labeled as “high” or there were “some concerns” for multiple domains that markedly lowered the confidence in the results. For non-randomized studies, we used the Risk of Bias in Non-Randomized studies – of Interventions (ROBINS-I) assessment tool [39]. This tool evaluated various types of bias that may occur in the process of conducting the studies pre-, at- and post-intervention domains. The judgment for each domain was either having “low”, “moderate”, “serious”, “critical” or “no information”. Similar to the RoB2, the overall risk of bias for each study was marked based on the presence of at least one higher risk item in one of the domains. For example, the overall risk of bias in a study was judged at “serious risk of bias” if at least one domain was labeled as “serious” given that there were no higher risk ratings in any of the domains such as “critical”. “No information” response was used when there was no clear indication that the risk of bias was at serious or critical and the information was lacking in one or more of the domains. Two authors (R.B. and A.M.) assessed the risk of bias and if disagreement occurred even after discussion between them, a third coder was invited to reach to conclusion.

## Results

### Study selection findings

**Fig. 1:**
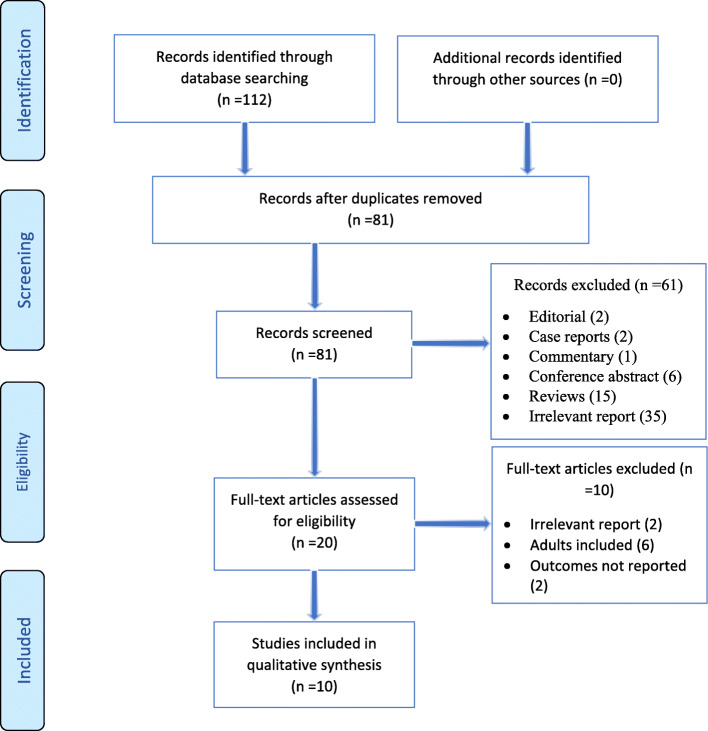
Flow chart depicting the method of study selection

In our review, 112 records were identified using our search strategy. Overall, 10 relevant studies were included in the current systematic review evaluating the efficacy of BCS in children with CRS. Figure 1 illustrates the study selection process.

### Study characteristics

The summarized characteristics of each study are shown in Table 1. A total of 263 patients were included. The sample sizes ranged from 12 to 42. The majority of the studies were prospective cohort, with two interventional controlled trials alongside. The duration of follow-ups ranged from 4 months to 5 years. Maxillary sinus, among all paranasal sinuses, was the predominant sinus being treated with BCS in children.

**Table 1:** Summary of the included studies

Study	Design	n^d^	Mean Age (Age range), years	Sinuses dilated via balloon	Inclusion	Adjunct procedure(s)	Follow-up time	Efficacy (by SN-5) Mean (SD)	Efficacy (by others) Mean (SD)	Revision, antibiotics use and AEs
**Ramadan HH et al. 2010a** [35]	**Prospective interventional non-randomized**	**30 (55 sinuses)**	**6.6 (2–11)**	**48 Maxillary 2 Frontal 5 Sphenoid**	**Chronic sinusitis (> 3 months or 6 episodes per year) AND 2 failed courses of antibiotics AND positive CT scan**	**Adenoidectomy (**n **= 17)**	**52 weeks**	**Preop: 4.2, Postop: 3;** **24 (80%) improved**^a^	**NR**	**Second intervention by ESS (2);** **AEs: NR**
**Ramadan HH et al. 2010b** [36]	**Prospective single arm cohort**	**24 (56 sinuses)**	**6.5 (2–11)**	**50 Maxillary 2 Frontal 4 Sphenoid**	**Refractory CRS which failed medical treatment AND evidence of CRS on CT scan**	**Adenoidectomy (**n **= 15); Ethmoidectomy (**n **= 6)**	**52 weeks**	**Preop: 4.9 (1.14), Postop: 2.95 (1.35);** **21 improved**^a^	**NR**	**AEs: None**
**Ramadan HH et al. 2012** [28]	**Prospective single arm cohort**	**26 (33 sinuses)**	**7.7 (4–12)**	**33 Maxillary**	**Chronic sinusitis (> 3 months or 6 episodes per year) after failed adenoidectomy AND positive CT findings.**	**Ethmoidectomy (n = 4); Maxillary antrostomy (n = 3); Revision adenoidectomy (**n **= 2)**	**52 weeks**	**Preop: 4.6 (0.9), Postop: 3 (1.2);** **21 (81%) improved**^a^	**NR**	**AEs: None**
**Thottam PJ et al. 2012** [30]	**Retrospective, two group cohort blinded**	**15 (40 sinuses)**	**9.3 (3–17)**	**30 Maxillary 10 Frontal**	**Refractory CRS (at least 90 days despite medical therapy) AND CT confirmed uncomplicated CRS with the presence of maxillary +/− frontal sinus disease.**	**Ethmoidectomy**	**> 4 months (average of 37 weeks)**	**SN-5: NR;** **12 (80.0%) Improved**^b^	**NR**	**Second intervention by ESS (1);** **Off Antibiotic: 11 (73.3%);** **AEs: None**
**Wang F et al. 2015** [29]	**Prospective interventional non-randomized controlled**	**42**	**9.3 (7–12)**	**Maxillary and Frontal**^c^	**Diagnosis of CRS according to EPOS 2012. Refractory CRS (defined as at least 2 years despite medical therapy)**	**Adenoidectomy**	**1 year**	**Preop: 4.3 (0.9), Postop: 2.9 (0.8);** **39 (92.9%) improved**^a^	**VAS** **Preop: 5.2 (1.4),** **Postop: 3.1 (1.6)**	**AEs: periorbital swelling (1)**
**Thottam PJ et al. 2016** [31]	**Retrospective, two group cohort**	**13 (34 sinuses)**	**9.3 (3–18)**	**26 Maxillary 8 Frontal**	**Refractory sinus symptoms despite medical treatments AND CT scan confirmed uncomplicated CRS and the presence of maxillary sinus disease with a Lund-Mackay score greater than 5.**	**Ethmoidectomy**	**2 years**	**SN-5: NR;** **10 (76.9%) Improved**^b^	**NR**	**Second intervention by ESS (1);** **AEs: NR**
**Soler ZM et al. 2017** [24]	**Prospective single arm cohort**	**33**	**6.6 (2–12)**	**Maxillary, Frontal and Sphenoid**^c^	**Diagnosis of CRS according to EPOS12, positive CT findings and disease resistant to medical treatments.**	**Adenoidectomy (**n **= 19); Inferior turbinate reduction (n = 6); Ethmoidectomy (n = 4); Tonsillectomy (**n **= 5); Others (**n **= 4)**	**6 months**	**Preop: 4.8 (1.2), Postop: 1.7 (0.8);** **32 (97%) improved**^a^	**NR**	**Second intervention (0);** **AEs: None**
**Liu J et al. 2017** [25]	**Prospective single arm cohort**	**30 (61 sinuses)**	**10.2 (6–15)**	**38 Maxillary 9 Frontal 14 Sphenoid**	**Confirmed by positive Nasal endoscopy and CT scan AND failed Medical management for at least 3 months**	**None**	**12 months**	**Preop: 23.04 (12.47), Postop: 1.07 (2.11)**	**Lund-Mackay score Preop: 12.29 (6.23), Postop: 1.39 (2.47);** **VAS Preop: 17.86 (7.59),** **Postop: 0.57 (1.60);** **Endoscopic score of Lund-kennedy preop: 6.69 (3.07),** **postop: 0.36 (1.89)**	**AEs: synechia (3)**
**Gerber ME et al. 2018** [32]	**Randomized controlled blinded study**	**12**	**7**	**Maxillary**^c^	**CRS with at least 12 weeks of two symptoms AND refractory to medical treatments AND recurrence (4 or more episodes per year) AND evidence of rhinosinusitis on CT scan.**	**Adenoidectomy with maxillary sinus irrigation (all patients)**	**12–18 months**	**Preop: 3.62 (1.24), Postop: 2.40 (1.11)**	**NR**	**NR**
**Zalzal HG et al. 2019** [33]	**Retrospective single arm cohort**	**38**	**6.76**	**Maxillary**^c^	**CRS with at least 90 days of 2 or more of symptoms, or endoscopic signs AND failure of medical treatment defined by EPOS 2012 AND previous adenoidectomy AND Lund-Mackay score of at least 5 AND maxillary sinus involvement.**	**None**	**5 years**	**SN-5: NR**	**NR**	**Second intervention by ESS: 5 (13.1%);** **Off antibiotic: 26 (68.4%)**

*AEs* Adverse events

*CRS* Chronic rhinosinusitis

*CT* Computed tomography

*ESS* Endoscopic sinus surgery

*EPOS 2012*: European position paper on rhinosinusitis and nasal polyps 2012

*NR* Not reported

*SN-5* Sino-Nasal 5 quality of life scale

*VAS* Visual analogue scale

^a^(0.5 decrease or more in SN-5 score) described by Kay and Rosenfeld

^b^Improvement were defined as a decrease in the total complaint score of 1 or more symptoms. Symptoms are facial pain, sinus congestion, postnasal drip, rhinorrhea, headache, and low-grade fever

^c^Number of sinuses dilated via balloon sinuplasty was not specified in the included sample

^d^Number of children underwent balloon sinuplasty and included in the analysis of the respective study

### Primary outcome (quality of life and symptoms improvement)

QoL and overall symptoms improvement are summarized in Table 1. The included studies examined subjective feedback from parents of children who underwent balloon sinuplasty. Evaluation of short-term follow-up results (6 months or less) showed that balloon sinuplasty had better outcomes than baseline results. Soler ZM et al. reported a significant improvement in SN-5 (mean score = 1.7 postoperatively vs. mean score = 4.8 preoperatively) [24]. In a prospective study conducted by Liu J et al., VAS score was significantly improved after the procedure (preop VAS score = 17.86 ± 7.59 vs. post op VAS score = 1.68 ± 3.53) [25]. Additionally, findings of the two experimental controlled trials supported the aforementioned findings [29, 32].

QoL and other pertinent measures were reported for the long-term follow-up (12 months or more). According to longitudinal studies, SN-5 results of the long-term follow-up showed a significant improvement in the score with sustained results [25, 28, 29, 31, 32, 35, 36]. According to a long-term evalution by VAS, a dramatic drop in the score was found in two cohorts of patients after a 1 year follow-up [25, 29]. In addition, long-term examination revealed that balloon sinuplasty had greater and more sustained improvement after 1 year (6 months postoperative VAS score of 1.68 ± 3.53 vs. 1-year postoperative score of 0.57 ± 1.60) [25]. Results of the two experimental controlled trials supported the aforementioned findings [29, 32].

In addition to the statistical significance of the results, the clinical significance was determined as described by Kay and Rosenfeld [1]. The overall clinical improvement was defined as a decrease of 0.5 or more in SN-5. Up to 97% of patients experienced an overall clinical improvement in QoL [24, 28, 29, 35].

To objectively evaluate patients who underwent sinuplasty, the Lund-Mackay score and Lund-Kennedy endoscopic score were assessed in a single study. The Lund-Mackay scale, more widely used in CRS, showed a significant improvement in CT findings, with a preoperative mean score of 12.29 compared to 1.39 postoperatively. Furthermore, the Lund-Kennedy score, measured by nasal endoscopy, improved from 6.69 to 0.36 [25].

### Secondary outcomes

The rate of second intervention was described in five studies [24, 30, 31, 33, 35]. A large study, in which 157 sinuses were dilated, had a success rate of 100%, i.e., no additional surgical intervention [24]. However, Zalzal HG et al. reported the highest failure rate (13.1%) [33]. The remaining reports had low failure rates at follow-up (less than 8%) [30, 31, 35]. Use of antibiotics at follow-up was mentioned in two reports [30, 33]. Zalzal HG and his colleagues reported that the vast majority of patients treated with the balloon technique (nearly 70%) were not found to take any course of sinusitis-indicated-antibiotics during their 5 years follow-up, while the remaining children in the same cohort required only a single course of antibiotic during the same period [33].

### Adverse events

While the majority of studies reported no complications , four patients experienced minor complications. Synechiae was the most commonly reported side effect of sinuplasty (*n* = 3), and all cases were treated by adhesiolysis at subsequent hospital visits [25]. One patient developed periorbital swelling, which was treated conservatively and resolved within a week [29].

### Risk of bias assessment

Table 2 shows the risk of bias in the included studies. There were nine non-randomized studies and one randomized trial included in the review. As we used the appropriate tools to evaluate the studies, detailed criteria for each item were modified to better serve our outcome measures. We evaluated the non-randomized studies using the ROBINS-I tool and the results are shown in Table 2. Most of the studies had moderate risk of bias mainly due to confounding bias and bias in selecting participants into the study [24, 25, 28–30, 35, 36]. Two studies had serious risk of bias due to a bias in the selection of participants since they were retrospective study designs [31, 33]. For the randomized study [32], we used the RoB 2 tool to assess the risk of bias (Table 2). The study was judged with some concerns since details of the randomization process were insufficient; however, baseline differences between the two groups were addressed. Also, assessment of the outcome as the parents of children were filling the questionnaires could have been influenced by their knowledge of receiving the balloon dilatation.

**Table 2:**
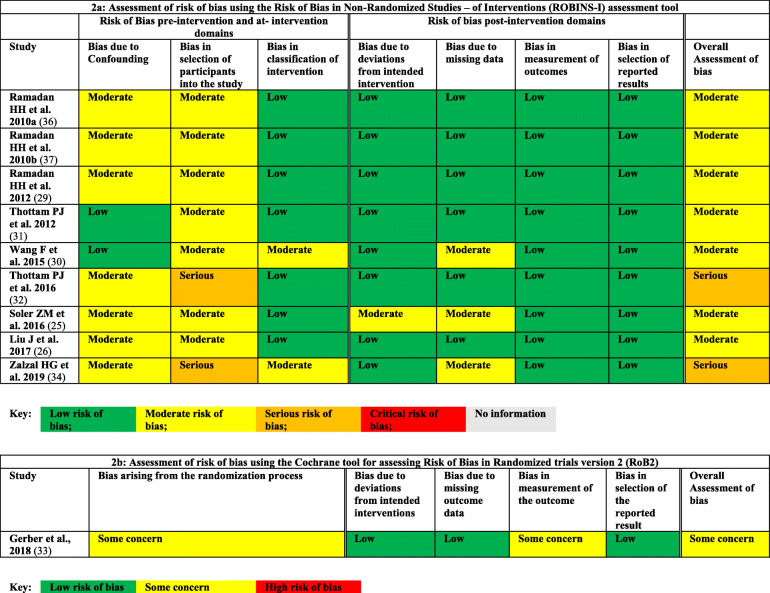
Risk of bias of the included studies using the Cochrane Collaboration’s Tools for risk of bias assessment

## Discussion

BCS is a relatively new and promising technique, particularly for children, owing to its noninvasive nature. Given the growing number of published studies and reported complications, the goal of the current review was to comprehensively elucidate the efficacy and side effects of this technqiue. Studies of the impact of BCS on QoL in pediatric CRS were aggregated in a previously published meta-analysis [34]. The previous meta-analysis had a restricted search strategy and the analysis was based on 4 observational based studies. We expanded on the latest review, adding more studies including intervention-based trials. We also evaluated adverse events, antibiotic usage and revision rate, in addition to QoL and overall symptoms improvement.

Similar to what was reported for adult CRS patients [27], this systematic review demonstrated that use of BCS to treat CRS in pediatric populations has a favorable impact on QoL. We found significant improvement in SN-5 score in short- and long-term follow-ups. In addition, the positive effect of BCS on QoL was sustained, and was further enhanced in long-term follow-ups [25].

Adenoidectomy, at the present time, is the first surgical option in pediatric CRS. A study carried out by Ramadan et al. showed that higher number of older children who underwent BCS, with adenoidectomy or standalone BCS, experienced an improvement in QoL than patients who underwent adenoidectomy alone [35]. Thus, BCS can potentially increase the success rate of adenoidectomy and might be an effective alternative for pediatric patients who had persistent symptoms after adenoidectomy [28].

BCS may be performed as a standalone procedure, or in certain cases, with concurrent sinonasal procedures, such as septoplasty, adenoidectomy, or inferior turbinate reduction. Although our study showed a high success rate of BCS, the concurrent or prior adenoidectomy procedure posed considerable limitations in demonstrating the efficacy of standalone balloon sinuplasty. Interestingly, Soler and his group adjusted for numerous confounding factors, including adjunctive procedures, and found that BCS was efficacious by itself [24]. The same study showed no clear difference in SN-5 score between a group who underwent BCS alone and another group that received BCS with concurrent procedures. In fact, children who received additional procedures required longer recovery times [24]. Therefore, BCS could be offered as standalone modality to improve the QoL. Ramadan et al., on the other hand, showed that children who underwent BCS and adenoidectomy had 91% improvement in their SN-5 score post operatively, compared to 85% for those who underwent BCS alone [36]. However, no hypothetical work-up has been carried out to detect any statistically significant difference. Thus, a head-to-head comparison of adenoidectomy versus standalone BCS on a larger scale is warranted.

Based on existing literature, BCS, in comparison to ESS, works by widening the normal sinus opening by balloon inflation without needing to change the normal sinus anatomy or causing damage to surrounding structures. Therefore, the probability of scarring, early revision requirement and facial developmental effects is low [40]. In our review, the rate of complications following BCS was very minimal, and only non-serious adverse events, such as synechia, were reported [24, 25, 29]. In addition, a second intervention by ESS was indicated in very few cases across the included studies. Therefore, BCS potentially can be recommended in children with CRS before ESS is planned. Another advantage of BCS over ESS is that a lower percentage of antibiotic usage among patients who underwent BCS was found in the long-term follow-ups [30, 33].

A group of researchers conducted an interventional controlled study to evaluate BCS. In parallel to the aforementioned findings, they found BCS to be effective in improving the QoL, but it had no additional value to the standard clinical practice, i.e. adenoidectomy with maxillary irrigation [32]. It is important to mention the limited number of samples, where only 12 were enrolled in the treatment group. Therefore, future randomized controlled trials with a larger sample numbers are required to conclusively demonstrate the potential merits of BCS.

Other findings in the current literature demonstrated the superiority and efficacy of BCS. For instance, sick leave duration was shorter in BCS compared with ESS [41]. Furthermore, Liu et al. examined the CT and endoscopic findings in patients who underwent BCS, and found a significant improvement in both modalities up to 1 year after operation [25].

BCS may be performed under general anesthesia in the operating room or in-office with local anesthesia. In a previous systematic review of adult patients, the QoL score was found to be significantly less in the operating room setting than following in-office BCS [27]. Soler ZM et al. compared BCS in a pediatric group between operating room and in-office settings; however, given the small sample, they were unable to make a reliable comparison [24].

Our review was limited by the number of included studies. Thus, a unifying conclusion was difficult to make. An important point to consider is the age at the time of intervention. Although the age range was presented in all of the studies, it is hard to determine the actual number of younger patients especially those less than 7 years old whose sinuses might have not been completely developed yet. Also, the lack of mention of adenoid size prior to removal makes it quite difficult to fairly judge the efficacy of BCS that was performed concurrent with adenoidectomy. Thus, the results should be interpreted cautiously, and future comparative studies with age-wise analysis and including adenoid size are currently warranted. In addition, most of the included studies were observational. Therefore, establishment of evidence-based practice, and enhancement of decision-making for using the BCS alone are hardly to be recommended at this moment. Future experimental randomized studies evaluating the BCS as standalone treatment for pediatric CRS are recommended. It must also be noted that BCS was assessed by a parent- reported QoL scale, which makes it difficult to extrapolate the efficacy of treatment. In the current literature, there is insufficient data to demonstrate efficacy; in addition, a remarkable heterogeneity was identified across the studies. Thus, the data were not aggregated in a meta-analysis.

## Conclusion

Balloon sinuplasty as a surgical treatment option is safe in pediatric CRS. However, future randomized controlled studies with larger sample size and long-term follow-up are needed. Such studies can further determine the efficacy of BCS in managing children with CRS.

## Data Availability

Not applicable.

## Abbreviations

CRS: Chronic rhinosinusitis
EPOS: The European Position Paper on Rhinosinusitis and Nasal Polyps
QoL: Quality of life
CT: Computed tomography
ESS: Endoscopic sinus surgery
BCS: Balloon catheter sinuplasty
SN-5: Sino-Nasal 5 quality of life scale
VAS: Visual analogue scale
RoB2: The revised risk of bias assessment tool
ROBINS-I: The risk of bias in non-randomized studies – of interventions
